# Supplementary material for: C/EBPB-dependent adaptation to palmitic acid promotes tumor formation in hormone receptor negative breast cancer
Source: Nat Commun. 2022 Jan 10;13:69. doi: 10.1038/s41467-021-27734-2 (PMC8748947; doi:10.1038/s41467-021-27734-2)

SUPPLEMENTAL INFORMATION

C/EBPB-dependent Adaptation to Palmitic Acid Promotes Tumor Formation in Hormone Receptor Negative Breast Cancer

The PDF file includes:

Supplementary Figures

Supplementary Figure 1

Supplementary Figure 2

Supplementary Figure 3

Supplementary Figure 4

Supplementary Figure 5

Supplementary Figure 6

Supplementary Tables

Supplementary Table 1 List of genes included in the targeted sequencing of PM/ER$^-$/PR$^-$

Supplementary Table 2. Antibody panel used for mass cytometry analysis

Supplementary Table 3. PCR Primer sequences used for barcode amplification

Supplementary Table 4. Material and resources table

Uncropped blots from Supplementary Figure 4 a-c

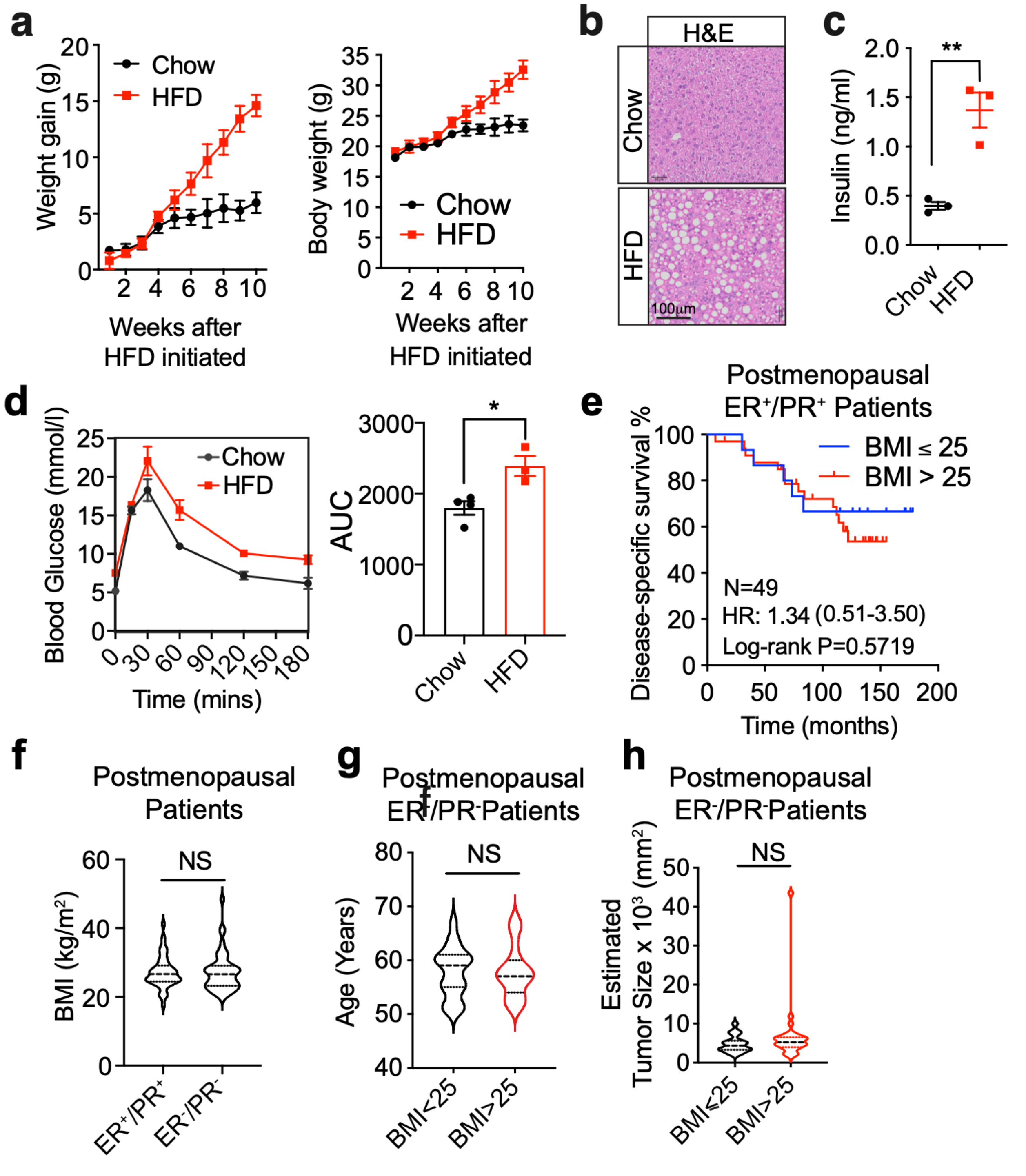

Supplementary Figure 1. a Body weight gain and absolute body weight of HFD and chow-fed mice before implantation of tumors. The measurement of animal body weight was started at six weeks of age and recorded weekly. For each time point, data is represented as mean ± SEM,

n=4 per group. **b** Hematoxillin and Eosin (H&E) stained tissue sections of livers from HFD and chow-fed mice. After ten weeks of HFD or chow diet feeding mice were sacrificed and and livers were harvested for H&E staining. Histological analysis showed increased liver steatosis in mice from the HFD group compared to mice from the chow group. **c** Concentration of fasting plasma insulin in mice. Concentrations were determined by ELISA using overnight fasted blood samples collected from female C57BL/6J mice fed an HFD or chow diet for ten weeks (n=3 per group, P = 0.0059) **d** Oral glucose tolerance test performed on mice fed an HFD (n=3) or chow (n=4) diet for ten weeks. Blood glucose concentrations were measured at 0, 15, 30, 60, 120 and 180mins following glucose administration by oral gavage. For each time point, data is represented as mean ± SEM, P=0.0151. AUC = area under the curve. **e** Kaplan-Meier curves display disease specific survival for postmenopausal and ER^+/PR^+ patients (n=49) with high (red, BMI > 25) or low (blue, BMI ≤ 25) BMI. Log-rank (Mantel-Cox) P value is denoted for difference in disease specific survival. The analysis showed no significant difference between the groups. **f** Distribution of BMI in postmenopausal ER^+/PR^+ (n=49) and ER^-/PR^-(n=48) patients. **g-h** Distribution of postmenopausal ER^-/PR^- patients' age (BMI > 25 n=29, BMI ≤ 25 n=19) (**g**) and estimated tumor size (BMI > 25 n=28 *one patient missing value, BMI ≤ 25 n=19) (**h**) in high and low BMI groups. The estimated tumor size was calculated by multiplying the largest diameter by its perpendicular. For **c-d**, statistical significance determined with unpaired, two-tailed Student's t-test. For **f-h** a two-tailed Kolmogorov-Smirnov test was used for statistical testing. (NS, P value > 0.05; *, P value < 0.05; **, P value < 0.01). Source data are provided as a Source Data file.

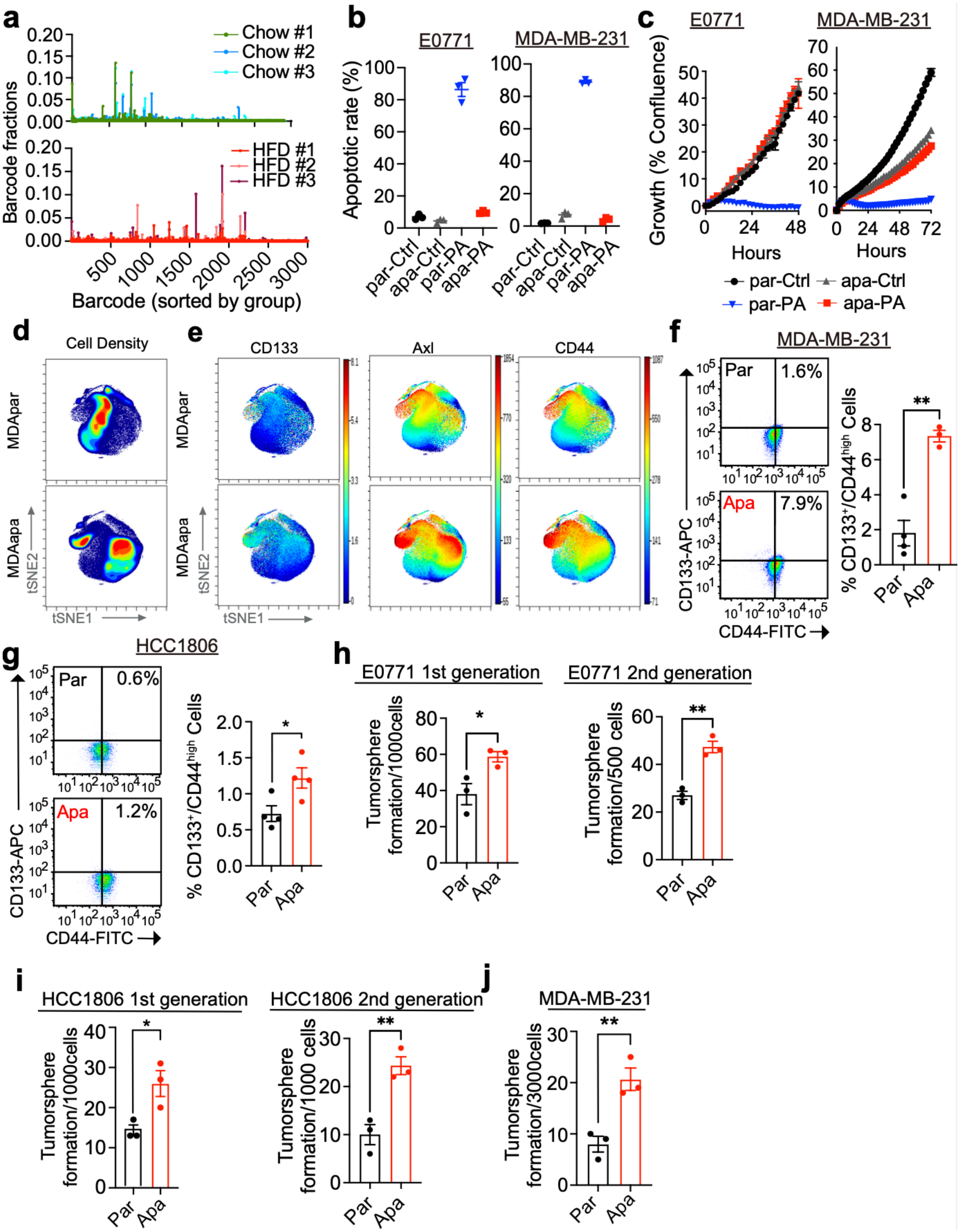

a
Chow #1
Chow #2
Chow #3
HFD #1
HFD #2
HFD #3
Barcode fractions
Barcode (sorted by group)
b
E0771
MDA-MB-231
Apoptotic rate (%)
par-Ctrl
apa-Ctrl
par-PA
apa-PA
c
E0771
MDA-MB-231
Growth (% Confluence)
Hours
par-Ctrl
apa-Ctrl
par-PA
apa-PA
d
Cell Density
MDApar
MDAapa
tSNE2
tSNE1
e
CD133
Axl
CD44
f
MDA-MB-231
Par
1.6%
Apa
7.9%
CD133-APC
CD44-FITC
% CD133+/CD44high Cells
g
HCC1806
Par
0.6%
Apa
1.2%
h
E0771 1st generation
E0771 2nd generation
Tumorsphere formation/1000cells
Tumorsphere formation/500 cells
i
HCC1806 1st generation
HCC1806 2nd generation
Tumorsphere formation/1000 cells
j
MDA-MB-231
Tumorsphere formation/3000cells

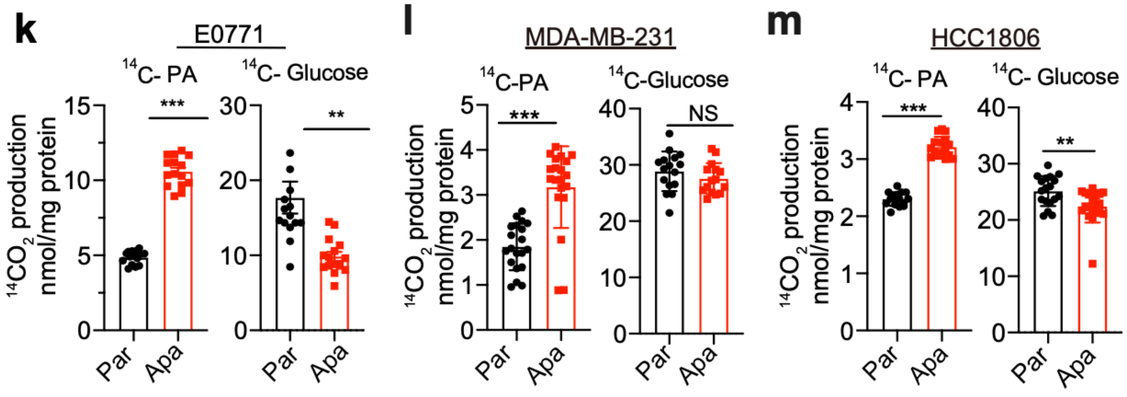

Supplementary Figure 2. a Barcode distribution of all replicates of tumors derived from chow and HFD-fed mice. The x axis of the histograms is barcode ID which were sorted by group and each bar represents one unique barcode. **b** Apoptotic rate of parental and adapted E0771 and MDA-MB-231 cells that were treated with PA (500 μM for E0771 and 400 μM for MDA-MB-231) and vehicle (Ctrl) for 48hrs. Data are represented as mean ± SEM of three replicates. **c** Proliferation assay of parental and adapted E0771 and MDA-MB-231 cells that were exposed to 400 μM (for MDA-MB-231) or 500 μM (for E0771) PA and vehicle (Ctrl). For each time point, data are represented as mean ± SEM of n=4 MDA-MB-231 and n=6 E0771 replicates. **d** Representative contour plots of mass cytometry data colored by density of cells showing the changes between parental and adapted MDA-MB-231 cells. Color code represents the cell density from low (blue) to high (red). **e** Representative tSNE plots of single parental and adapted MDA-MB-231 cells colored by expression of CD133, Axl and CD44. **f-g** Flow cytometry results of $CD133^+/CD44^{high}$ cells population in parental and adapted MDA-MB-231 P=0.0018 (**f**) and HCC1806 P=0.0326 (**g**) cells. n=4 replicates for each condition (one outlier in MDA-MB-231apa group was excluded from the quantification by Grubbs' outlier test). **h-j** Tumorsphere formation assay and serial tumorsphere propagation assay of parental and adapted E0771 (P=0.0338) (**h**), HCC1806 (P=0.0285) (**i**) and MDA-MB-231 (P=0.009) (**j**) cells. The serial tumorsphere propagations were performed with the dissociated primary tumrospheres of E0771 cells (P=0.0024) (**h**) and HCC1806 cells (P=0.0068) (**i**). Three replicates for each condition. **k-m**

Comparison of fatty acid and glucose oxidation assays between parental and adapted E0771 (n=15, P_{PA}<0.0001, $P_{glucose}$=0.0014) (**k**), MDA-MB-231 (n=20, P_{PA}<0.0001; n =16 for par, n=15 for apa, $P_{glucose}$=0.2595) (**l**) and HCC1806 (n=16, P_{PA}<0.0001; n =18 for par, n=20 for apa, $P_{glucose}$=0.0051) (**m**) cells. For **f-m**, date presented as mean ± SEM, and statistical significance determined with unpaired, two-tailed Student's t-test. (NS, P value > 0.05; *, P value < 0.05; **, P value < 0.01; ***, P value < 0.001). Source data are provided as a Source Data file.

a

Sample	Final library size after filtering (PE reads)	% Mito reads[a]	TSS[b] enrichment score	FRiP[c] score
E0771 HFD - rep 1	44374322	2.9	17.61	0.6
E0771 HFD - rep 2	43550673	3.6	18.17	0.62
E0771 HFD - rep 3	49268227	3.4	17.76	0.58
E0771 Chow – rep 1	49749601	2.5	16.76	0.6
E0771 Chow – rep 2	53174112	2.8	16.82	0.6
E0771 Chow – rep 3	47556103	6.4	17.42	0.61
E0771 Chow – rep 4	43695078	4.2	17.11	0.6

[a] Reads mapped to the mitochondrial genome
[b] Transcription start site
[c] Fraction of reads in called peaks

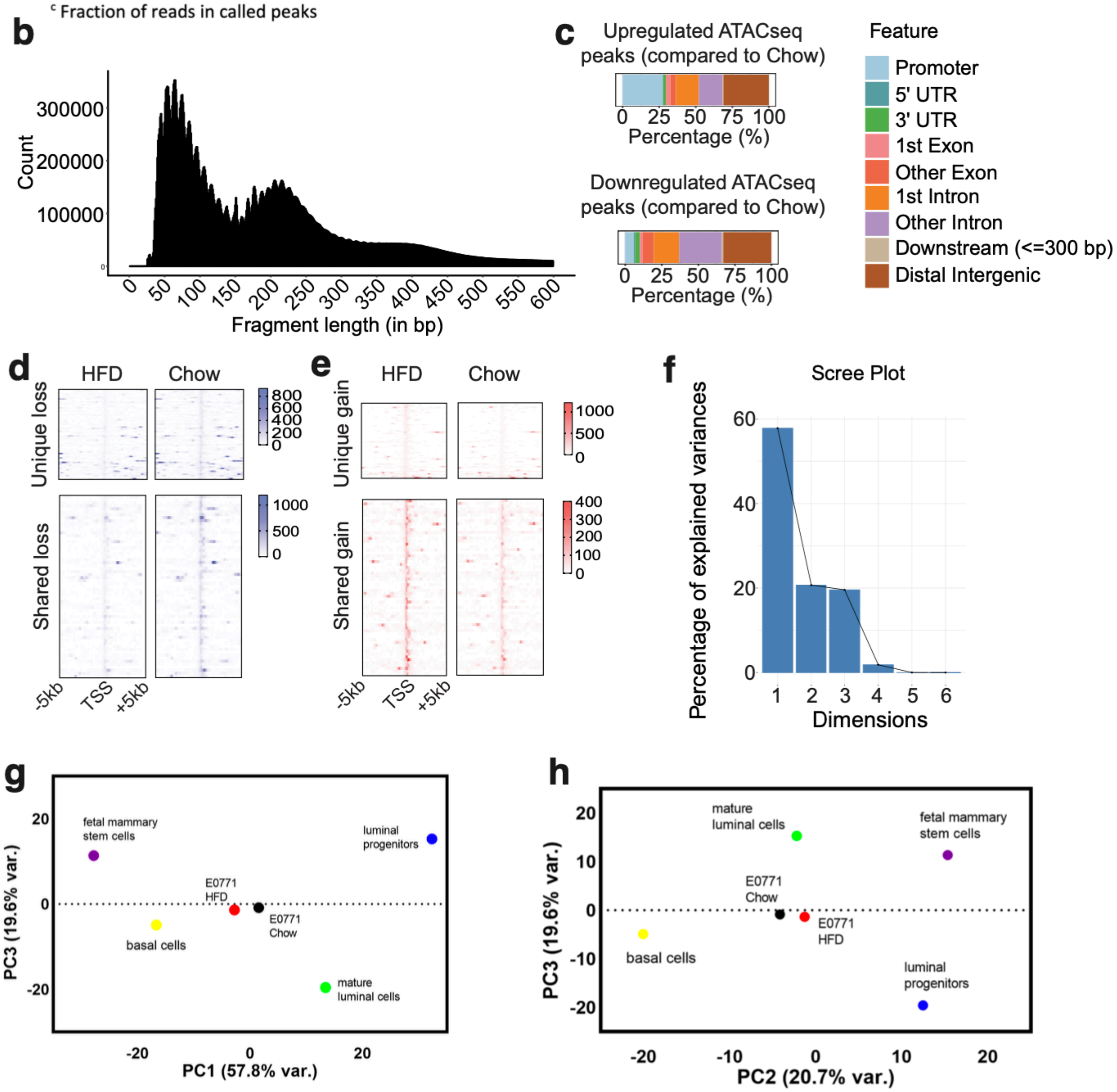

i **j**

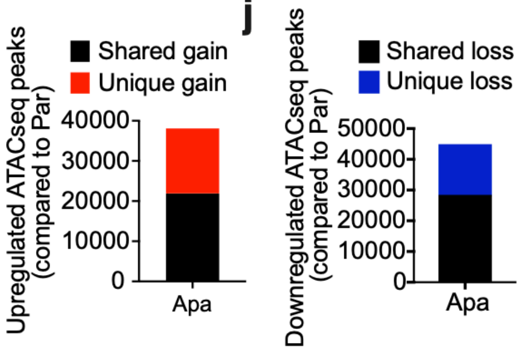

k

Sample	Final library size after filtering (PE reads)	% Mito reads[a]	TSS[b] enrichment score	FRiP[c] score
MDAapa – rep 1	34846799	22.7	22.41	0.69
MDAapa – rep 2	23764672	17	21.21	0.65
MDAapa – rep 3	42777985	18.1	20.7	0.63
MDApar – rep 1	72795834	9.1	22.95	0.72
MDApar – rep 2	37940089	8.7	22.44	0.71
MDApar – rep 3	49305950	9.6	22.74	0.71

[a]Reads mapped to the mitochondrial genome
[b]Transcriptional Start Site
[c]Fraction of reads in called peaks

l

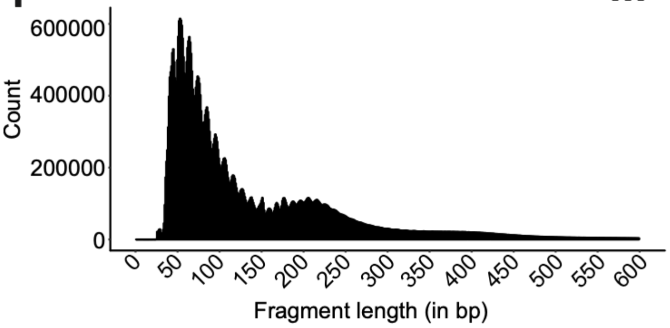

m

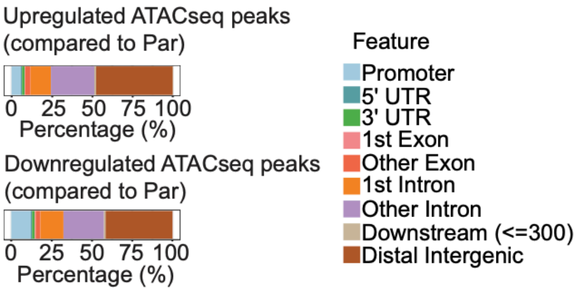

n

E0771 ATAC (Unique gain in HFD)

Motifs		p-value	% of targets sequences with motif	% of background sequences with motif
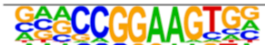	GABPA	1e-3	95.08	79.84
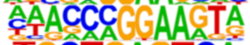	EHF	1e-3	93.44	80.58
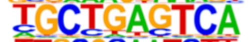	MAF	1e-2	86.89	73.48
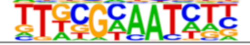	C/EBP	1e-2	70.49	54.98

o MDA ATAC (top 5% of unique gain in Apa)

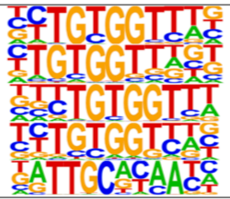

Motifs		p-value	% of targets sequences with motif	% of background sequences with motif
	RUNX1	1e-55	88.03	63.44
	RUNX2	1e-39	87.55	67.48
	RUNX3	1e-36	88.66	69.99
	PEBB	1e-21	81.26	66.10
	C/EBPB	1e-12	71.52	59.25

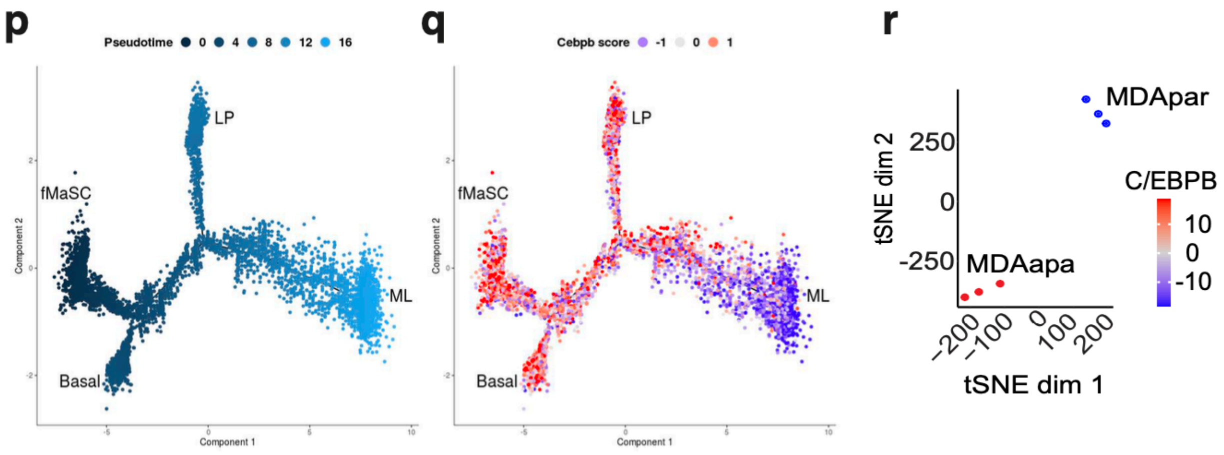

Supplementary Figure 3. a Final library size after filtering and quality metrics of the ATACseq libraries reported in **Figure 3a** and **b**, including percentage of initial library reads before filtering mapped to the mitochondrial genome and signal-to-noise ratios of the final filtered libraries (transcription start site enrichment scores and fraction of called peaks in the library reads). **b** Fragment size distribution of a representative ATACseq library reported in **Figure 3a** and **b**, indicative of a nucleosomal laddering pattern typical of an ATACseq library. **c** Genome annotation of the differential ATACseq peaks reported in Figure 3a and b with respect to their position using ChIPseeker. Promoters are defined as -1000 and +100 bp from the nearest transcription start site (TSS), distal intergenic regions as beyond -1000 bp from this start site and downstream as ≤ +300 bp from the nearest transcription end site. **d** Heatmaps of the significantly upregulated ATACseq peaks (unique and shared gain) in E0771 HFD relative to chow as reported in Figure 3a. Each row represents one peak in relation to a ±5kb window centered around the TSS and peaks are ranked in a descending order according to the fold change (HFD/Chow). **e** Heatmaps of the

significantly downregulated ATACseq peaks (unique and shared loss) in E0771 HFD relative to chow as reported in Figure 3b. Each row represents one peak in relation to a ±5kb window centered around the TSS and peaks are ranked in an ascending order according to the fold change (HFD/Chow). **f** Scree plot showing the percentage of variance of the data in Figure 3c explained by the first six principal components (PC). Note that the first three PCs capture more than 98% of the variance. **g-h** Biplots showing PC1 vs PC3, and PC2 vs PC3, respectively, of the E0771 *ex vivo* cells and different cell lineages along the mammary gland developmental trajectory as in Figure 3c**. i** Total number of significantly upregulated ATACseq peaks in MDAapa (N = 3) relative to MDApar (N = 3) using diffBind with an FDR < 0.05. Unique gain peaks refer to peaks identified only in the adapted condition, whereas shared peaks are peaks called in both conditions. **j** Total number of significantly downregulated ATACseq peaks in MDAapa relative to MDApar using diffBind with an FDR < 0.05. Unique loss peaks refer to peaks identified only in the parental condition, whereas shared peaks are peaks called in both conditions. **k** Final library size after filtering and quality metrics of the ATACseq libraries reported in Figure S3i and j, including percentage of initial library reads before filtering mapped to the mitochondrial genome and signal-to-noise ratios of the final filtered libraries (transcription start site enrichment scores and fraction of called peaks in the library reads). **l** Fragment size distribution of a representative ATACseq library reported in Figure S3i and j, indicative of a nucleosomal laddering pattern typical of an ATACseq library. **m** Genome annotation of the differential ATACseq peaks reported in Supplementary Figure 3i and j with respect to their position using ChIPseeker as Figure S3c. **n** Motif enrichment analysis using Homer and HOCOMOCO motifs in the unique gain ATACseq peaks in E0771 HFD as reported in Figure 3a. **o** Motif enrichment analysis using Homer and HOCOMOCO motifs in the top 5% of unique gain ATACseq peaks (ranked by fold changes Apa/Par) in MDAapa as reported in Figure S3i. **p** Pseudotime analysis of single-nuclei ATACseq of murine mammary cells at different developmental stages (GSE125523). **q** Motif enrichment of transcription factors C/ebpb in the open chromatin regions at each individual cell along the

mammary gland developmental trajectory is shown. fMaSC, fetal mammary stem cells; basal, adult basal cells; LP, luminal progenitors; and ML, mature luminal cells. **r** t-SNE clustering of individual MDApar and MDAapa replicates showing differential motif enrichment in transcription factors C/EBPB. For **n** and **o**, the p-values were determined using default binomial distribution in HOMER. Source data are provided as a Source Data file.

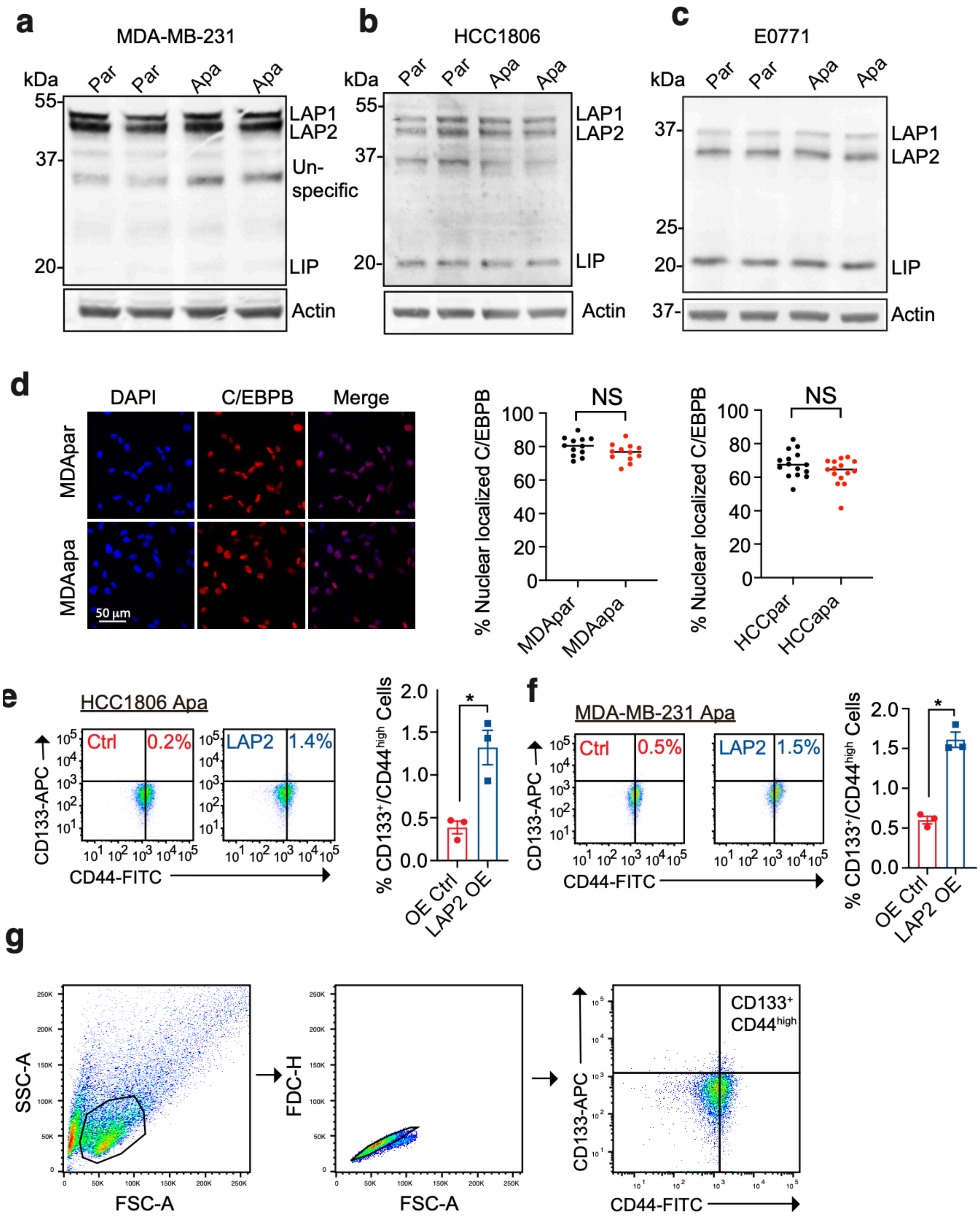
a
MDA-MB-231
kDa
Par
Par
Apa
Apa
55
37
20
LAP1
LAP2
Un-
specific
LIP
Actin
b
HCC1806
kDa
Par
Par
Apa
Apa
55
37
20
LAP1
LAP2
LIP
Actin
c
E0771
kDa
Par
Par
Apa
Apa
37
25
20
37
LAP1
LAP2
LIP
Actin
d
DAPI
C/EBPB
Merge
MDApar
MDAapa
50 μm
% Nuclear localized C/EBPB
NS
MDApar
MDAapa
HCCpar
HCCapa
e
HCC1806 Apa
Ctrl
0.2%
LAP2
1.4%
CD133-APC
CD44-FITC
% CD133+/CD44high Cells
OE Ctrl
LAP2 OE
f
MDA-MB-231 Apa
Ctrl
0.5%
LAP2
1.5%
CD133-APC
CD44-FITC
% CD133+/CD44high Cells
OE Ctrl
LAP2 OE
g
SSC-A
FSC-A
FDC-H
FSC-A
CD133-APC
CD44-FITC
CD133+
CD44high

Supplementary Figure 4. a-c Immunoblots of C/EBPB and Actin in parental and adapted MDA-MB-231 (**a**), HCC1806 (**b**) and E0771 (**c**) cell lines. Actin was used for the normalization. Western blots were repeated independently, three times for MDA-MB-231 and HCC1806, once for E0771. **d** Representative images of C/EBPB-immunofluorescent staining on MDApar and MDAapa cells. Quantification was calculated by the percentage of C/EBPB localized in the nucleus compared to the cytoplasm for MDA-MB-231 (12 cells from different regions were randomly picked) and HCC1806 (15 cells from different regions were randomly picked) parental and PA-adapted cell lines. **e-f** $CD133^{+}/CD44^{high}$ cells population in adapted HCC1806 (P=0.0121) (**e**) and MDA-MB-231 (P=0.0007) (**f**) cells overexpressing LAP2. Cells were stained by CD133-APC and CD44-FITC antibodies and measured by flow cytometry. Quantification data is shown as mean ± SEM of three replicates. **g** Gating strategy for flow cytometry analysis. FSC-A and SSC-A were used to exclude debris and dead cells, and FSC-A and FSC-H were used to exclude doublets. To gate the $CD44^{high}/CD133^{+}$ cell population, the median fluorescent intensity (MFI) of CD44-FITC was measured on control replicates. The average value of CD44-FITC MFI was then used to gate $CD44^{high}$ cells, and $CD133^{+}$ cells were gated according to the negative staining samples. For e-f, statistical significance determined with unpaired, two-tailed Student's t-test. (NS, P value > 0.05; *, P value < 0.05). Source data are provided as a Source Data file.

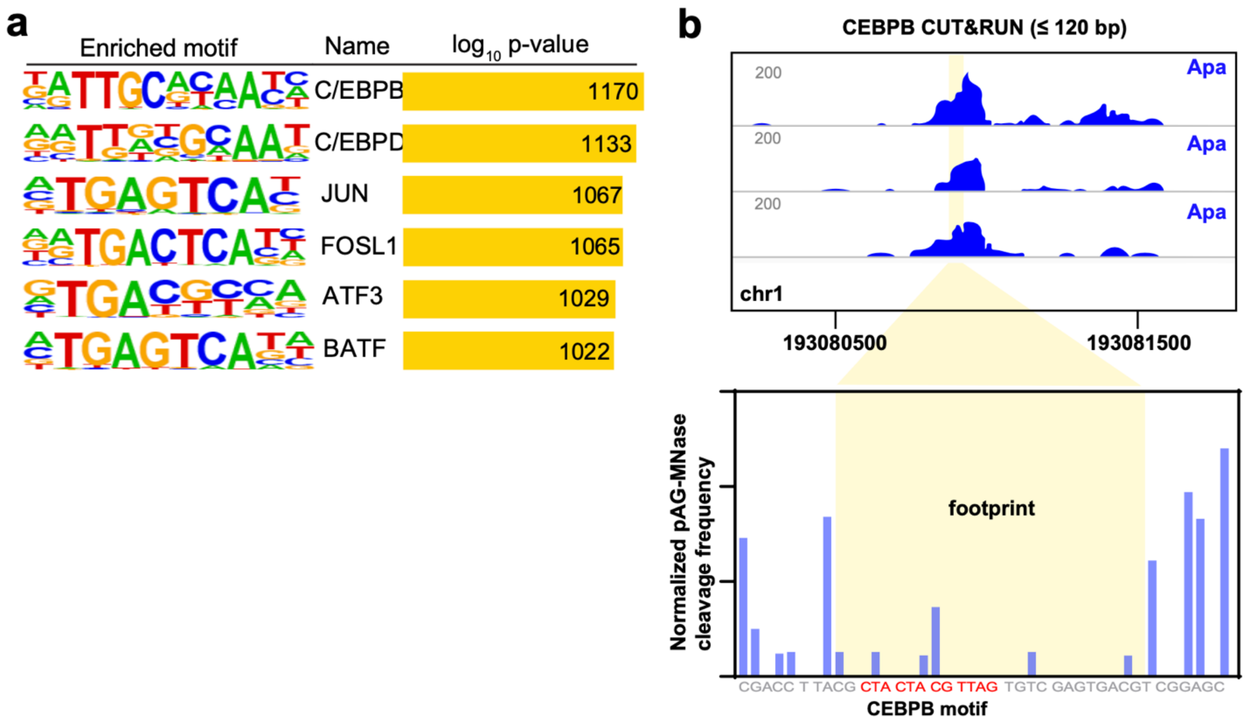

Supplementary Figure 5. a Motifs enriched in C/EBPB Cut&Run footprints in MDAapa cells. The p-values shown in the figure were reported by HOMER using HOCOMOCO motifs. **b** Single locus footprint analysis of C/EBPB Cut&Run experiments in adapted MDA-MB-231 cells. Upper panel shows representative genome browser tracks of C/EBPB Cut&Run signal in the specified region in chromosome 1 (chr1). Lower panel shows the total normalized pA/G-MNase cut frequency of the three biological replicates at each nucleotide around the *C/EBPB* motif within the identified footprint in the specified region.

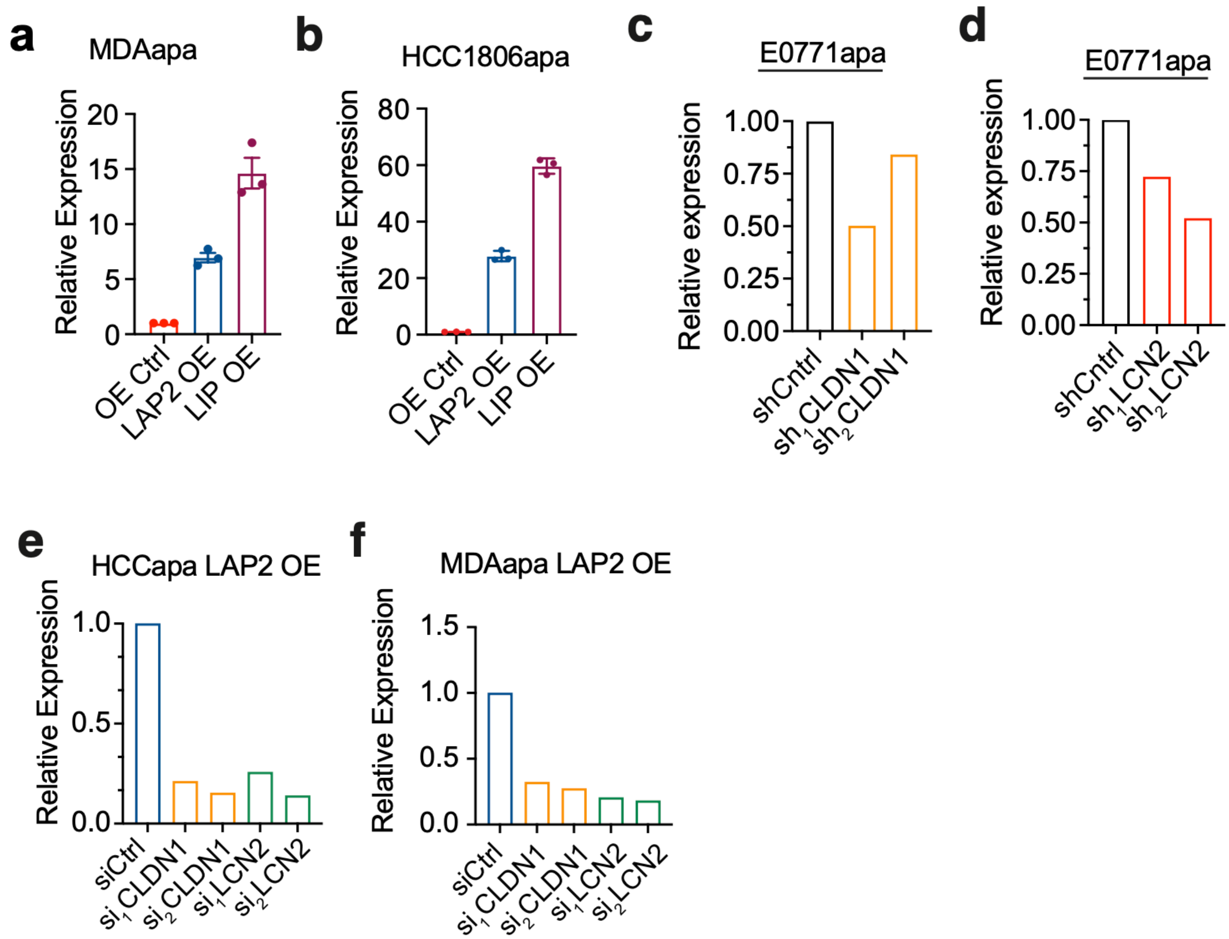

Supplementary Figure 6. a-b RT-qPCR was used to measure changes in the expression of *C/EBPB* upon the overexpression of *C/EBPB LAP2* and *LIP* isoforms on adapted MDA-MB-231 (**a**) and HCC1806 (**b**) cells. The relative expression is shown as relative fold change over control cells. Data shown as mean ± SEM of three independently repeated experiments. **c-d** RT-qPCR was used to measure efficiency of *Cldn1* (**c**) and *Lcn2* (**d**) knockdown in adapted E0771 cells. Knockdown was performed by using two independent shRNAs for each gene. **e-f** RT-qPCR was used to measure efficiency of *CLDN1* (yellow) *and LCN2* (green) knockdown relative to knockdown control (siCtrl, blue) in adapted HCC1806 (**e**) and MDA-MB-231 (**f**) cells. Knockdown was performed by using two independent siRNAs for each gene. Source data are provided as a Source Data file.

Supplementary Table 1. List of genes included in the targeted sequencing of PM/ER$^-$/PR$^-$

ABL1	CD79A	EPHB1	GRB2	MAP3K14	NBN	PTCH2	SUFU
ABL2	CD79B	EPHB4	GRB7	MAP3K2	NCOA2	PTEN	TBX22
ACVR2A	CDC6	EPHB6	GRID1	MAP3K3	NCOA3	PTK6	TBX3
AKT1	CDC73	ERBB2	GUCY1A2	MAP3K4	NF1	PTP4A1	TERT
AKT2	CDH1	ERBB3	H3F3A	MAP3K5	NF2	PTP4A3	TET2
AKT3	CDH2	ERBB4	HIST1H3B	MAP3K6	NFE2L2	PTPN11	TGFBR2
ALK	CDH20	ERCC2	HMGA2	MAP3K7	NGFR	RAB23	TNFAIP3
APC	CDH5	ERCC3	HNF1A	MAP3K8	NKX2-1	RAB25	TOP1
AR	CDK12	ERCC4	HOXA3	MAP3K9	NOTCH1	RAC1	TP53
ARAF	CDK4	ERCC5	HRAS	MAP4K3	NOTCH2	RAF1	TP63
ARFRP1	CDK6	ESR1	HSP90AA1	MAP4K4	NOTCH3	RARA	TP73
ARID1A	CDK8	ETV1	HSP90AB1	MAPK1	NOTCH4	RB1	TRAF2
ARID1B	CDKN1B	EZH2	ID1	MAPK10	NOV	REG4	TSC1
ARID2	CDKN2A	FADD	IDH1	MAPK7	NPM1	RET	TSC2
ASXL1	CDKN2B	FAM123B	IDH2	MAPK8	NRAS	RICTOR	TSHR
ATM	CDKN2C	FANCA	IGF1R	MAPK9	NTRK1	RNF43	U2AF1
ATR	CEBPA	FANCC	IGF2R	MCL1	NTRK2	ROBO1	USP9X
ATRX	CHD1	FANCD2	IKBKE	MDM2	NTRK3	ROBO2	VEGFA
AURKA	CHD1L	FANCE	IL7R	MDM4	OR5L1	ROS1	VHL
AURKB	CHEK1	FANCF	INHBA	MED12	PAK1	RPS6KB1	WHSC1L1
AXIN1	CHEK2	FANCG	INSR	MED12L	PAK3	RPTOR	WSB1
BAG4	CIC	FAS	IRS2	MED13	PALB2	RRM2B	WT1
BAP1	CKS1B	FBXO11	JAK1	MED29	PARP10	RSPO2	XPA
BCL11A	COL22A1	FBXW7	JAK2	MEN1	PAX5	RSPO3	XPC
BCL2	CREBBP	FGFR1	JAK3	MET	PAX9	RUNX1	XPO1
BCL2A1	CRKL	FGFR2	JUN	MITF	PBRM1	SETD2	YAP1
BCL2L1	CTCF	FGFR3	KAT6B	MLH1	PDGFRA	SF3B1	YWHAB
BCL2L2	CTNNB1	FGFR4	KDM5A	MLL	PDGFRB	SFTPA1	YWHAQ
BCL6	DAXX	FH	KDM5C	MLL2	PHF6	SHC1	YWHAZ
BCOR	DCUN1D1	FLT1	KDM6A	MLL3	PHGDH	SKP2	ZNF217
BIRC2	DDR2	FLT3	KDR	MPL	PHLPP2	SLIT2	ZRSR2
BIRC7	DDX11	FLT4	KEAP1	MRAS	PIK3C3	SMAD2	
BLM	DDX3X	FOXA1	KIT	MRE11A	PIK3CA	SMAD3	
BPTF	DICER1	FOXL2	KRAS	MSH2	PIK3CG	SMAD4	
BRAF	DNMT3A	FOXO1	LGALS7	MSH6	PIK3R1	SMARCA4	
BRCA1	DYRK1B	FOXP4	LTK	MST1	PIK3R2	SMARCB1	
BRCA2	E2F3	GAB2	MAFA	MTDH	PLCG1	SMO	
BRIP1	EEF1A2	GABRG1	MAP2K1	MTOR	POU1F1	SMURF1	
BUB1B	EGFR	GATA1	MAP2K2	MUTYH	PPM1D	SOCS1	
C11orf30	EIF5A2	GATA2	MAP2K4	MYB	PPP2R1A	SOX10	
CARD11	ELK3	GATA3	MAP2K5	MYC	PRDM1	SOX2	
CASP8	EP300	GATA6	MAP2K6	MYCL1	PRDM9	SOX9	
CBL	EP400	GNA11	MAP2K7	MYCN	PREX2	SPOP	
CCND1	EPHA3	GNAQ	MAP3K1	MYD88	PRG4	SRC	
CCND2	EPHA5	GNAS	MAP3K11	MYO3A	PRKCG	SRSF2	
CCND3	EPHA6	GPC5	MAP3K12	MYO5B	PRKCI	STAT3	
CCNE1	EPHA7	GPR124	MAP3K13	MYOC	PTCH1	STK11	

Supplementary Table 2. Antibody panel used for mass cytometry analysis

Isotope	Antigen	Cell Location	Epitope	Phenotype	Clone
168Er	Axl	Extracellular	Total	Stemness/EMT	1H12
160Gd	CD133	Extracellular	Total, Epitope 1	Stemness	AC133
173Yb	CD44	Extracellular	Total, Surface	Stemness	IM7
158Gd	E-cadherin	Extracellular	CD324/E-Cadherin	Epithelial	24E10
170Er	EGFR	Extracellular	Total EGFR	Epithelial organs/initiates MAPk, Akt and JNk signalling	AY13
143Nd	N-cadherin	Extracellular	CD325/N-Cadherin	Stemness/mesenchymal	8C11
156Gd	p38	Intracellular	p38[T180/Y182]	MAPK for stress response	D3F9
152Sm	pAkt	Intracellular	pAkt [S473]	PI3K pathway	D9E
176Yb	pCreb	Intracellular	pCREB [S133]	Transcription factor - stress and growth	87G3
151Eu	pEGFR	Intracellular	pEGFR [Y1068]	Activated EGFR	Y38
154Sm	pErk1/2	Intracellular	pT202/pY204	Branch of MAPK-Mek pathway	20A
175Lu	pHistone H3	Intracellular	pHistone H2A.X [Ser139]	Metaphase. Activated downstream of p38 or Erk1/2	HTA28
159Tb	pMAPKAPK2	Intracellular	pMAPKAPK2 [T334]	ERk1/2 activated protein downstream of p38. Response to stress	27B7
166Er	pNFKB	Intracellular	pNF-κB p65 [S529]	Transcription factor - mediator of inflammatory and immune responses	K10-895.12.50
162Dy	pPLCgamma	Intracellular	pPLCg2[pY759]	Mediator of inflammatory and immune responses	K86-689.37
150Nd	pRb	Intracellular	pRb[S807/S811]	G1 to S cell cycle phase	J112-906
172Yb	pS6	Intracellular	pS6[S235/S236]	Protein translation	N7-548
141Pr	pSHP2	Intracellular	Y580	RTK phosphatase promotes signaling of JAK/STAT, PI3K/Akt Ras/MAPK pathway	D66F10
153Eu	pStat1	Intracellular	Y704		4a
145Nd	pStat3	Intracellular	pY705		4/p
146Nd	pStat5	Intracellular	pY694		00047
149Sm	pStat6	Intracellular	Y641		18/P-stat6
163Dy	TGFbeta	Intracellular	Total		TW4-6H10
154Sm	Vimentin	Intracellular	Total	Mesenchymal	D21H3
167Er	YAP	Intracellular	CTD 379-407	Stemness Hippo	H9
172Yt	CC3	Intracellular	Cleavage at D175	Apoptosis	5A1E
164Dy	CK7	Intracellular	Total	Luminal marker	RCK105

Supplementary Table 3. PCR Primer sequences used for barcode amplification

	Sequence	Length
WS PCR Forward Primer	AATGATACGGCGACCACCGAGATCTACACACTGACTGCAGTCTGAGTCTGACAG	54
WS_Rev_Index_011	CAAGCAGAAGACGGCATACGAGATGTATCACGACGTGACTGGAGTTCAGACGTGTGCTCTTCCGATCTCTAGCACTAGCATAGAGTGCGTAGCT	94
WS_Rev_Index_013	CAAGCAGAAGACGGCATACGAGATAGCGTCTGATGTGACTGGAGTTCAGACGTGTGCTCTTCCGATCTCTAGCACTAGCATAGAGTGCGTAGCT	94
WS_Rev_Index_014	CAAGCAGAAGACGGCATACGAGATCAGCATGTCTGTGACTGGAGTTCAGACGTGTGCTCTTCCGATCTCTAGCACTAGCATAGAGTGCGTAGCT	94
WS_Rev_Index_015	CAAGCAGAAGACGGCATACGAGATGTACTCATCGGTGACTGGAGTTCAGACGTGTGCTCTTCCGATCTCTAGCACTAGCATAGAGTGCGTAGCT	94
WS_Rev_Index_016	CAAGCAGAAGACGGCATACGAGATTCTGCAGCTAGTGACTGGAGTTCAGACGTGTGCTCTTCCGATCTCTAGCACTAGCATAGAGTGCGTAGCT	94
WS_Rev_Index_017	CAAGCAGAAGACGGCATACGAGATACTGTACTCGGTGACTGGAGTTCAGACGTGTGCTCTTCCGATCTCTAGCACTAGCATAGAGTGCGTAGCT	94
WS_Rev_Index_018	CAAGCAGAAGACGGCATACGAGATCGACAGCTATGTGACTGGAGTTCAGACGTGTGCTCTTCCGATCTCTAGCACTAGCATAGAGTGCGTAGCT	94

Supplementary Table 4. Material and resources table

REAGENT or RESOURCE	SOURCE	IDENTIFIER
**Antibodies**		
Axl (application – TMA staining)	R&D Systems	AF154
C/EBPB	Santa Cruz	sc-7962
CD133 (application – TMA staining)	Miltenyi Biotec	130-090-422
Beta-actin	Invitrogen	PA1-183
E-cadherin-158Gd, Extracellular	Fluidigm	3158021A
Cleaved caspase 3-142Nd, Intracellular	Cell Signalling technology	Clone SA1E
CD44-173Yb, Extracellular	Fluidigm	3150018B
CD133-160Gd, Extra- and intracellular, conjugated in the lab	Miltenyi Biotec	130-090-422
Axl-168Er, Extracellular	BGB/creative biolabs	HPAB-0110-LS
CD133-APC	Invitrogen	17-1331-81
CD44-FITC	BioLegend	338803
pEGFR-151Eu, Intracellular, conjugated in the lab	abcam	ab32430
PCreb-176Yb, Intracellular	Fluidigm	3176005A
PAkt-152Sm, Intracellular	Fluidigm	3156002A
P38-156Gd, Intracellular	Fluidigm	3156002A
N-cadherin-143Nd, Extracellular	Fluidigm	3143016B
Keratin7-164Dy, Extracellular	BD	ab9021
EGFR-170Er, Extra- and intracellular	Fluidigm	3170009B
YAP-167Er, Intracellular, conjugated in the lab	Santa Cruz	sc-271134
Vimentin-154Sm, Intracellular	Fluidigm	3154014A
TGFβ-163Dy, Extra- and intracellular	Fluidigm	3163010B
PStat5-147Sm, Intracellular, conjugated in the lab	BD	562077
PStat3-145Nd, Intracellular, conjugated in the lab	BD	624084
PStat1-153Eu, Intracellular	Fluidigm	3153005A
PSHP2-141Pr, Intracellular	Fluidigm	3141002A
PS6-172Yb, Intracellular	Fluidigm	3172008A
PRb-150Nd, Intracellular	Fluidigm	3150013A
PNFKB-166Er, Intracellular	Fluidigm	3166006A
PMAPKAPK2-159Tb, Intracellular	Fluidigm	3159010A
pHistone H3-175Lu, Intracellular	Fluidigm	3175012A
pErk1/2-171Yb, Intracellular, conjugated in the lab	BD	624084
AF647 goat anti-mouse	Life Technologies	A21238
IRDye® 800CW Donkey anti-Rabbit IgG (H + L), 0.1 mg	Leicor	[P/N 926-32213], 0.1 mg
IRDye® 680RD Goat anti-Mouse IgG (H + L), 0.1 mg	Leicor	[P/N 925-68070], 0.1 mg
Mouse IgG isotype	Merck-Millipore	12-371

Anti-Histone H3 (mono methyl K4) antibody - ChIP Grade	Abcam	ab8895
Anti-trimethyl-Histone H3 (Lys27) Antibody	Merck	07-449
Rabbit IgG	Diagenode	C15410206
**Bacterial and Virus Strains**		
MAX Efficiency™ DH5α™ Competent Cells	Thermofisher Scientific	18258012
**Biological Samples**		
TMAs of breast cancer tissues	Haukeland University Hospital	
**Chemicals, Peptides, and Recombinant Proteins**		
Penicillin/streptomycin	Sigma	P-0781
Fetal bovine serum	Sigma	F-7524
DMEM	Sigma	D5671
RPMI1640	Sigma	R8758
BSA, fatty-acids free	Sigma	A7030
Palmitic Acid	Sigma	P5585
Phusion polymerase	NEB	M0530S
QIAquick Gel Extraction Kit	QIAGEN	28704
QIAGEN Plasmid Plus Maxi Kit	QIAGEN	12965
QIAquick PCR Purification Kit	QIAGEN	28104
E.Z.N.A. Tissue DNA Kit	Omega Bio-tek	D3396
Opti-MEM	Thermo Fisher	31985070
Lipofectamine 2000	Invitrogen	11668019
Polybrene Infection / Transfection Reagent	Sigma	TR-1003-G (1 ML)
Puromycin dihydrochloride from Streptomyces alboniger	Sigma	P8833-100MG
Annexin V, Alexa Fluor™ 488 conjugate	Thermo Fisher	A13201
Propidium Iodide	Sigma	P4864
Trypsin	Sigma	T4049
BSA	Sigma	A9647
Accutase	Sigma	A6964
DAKO EnVision+ System- HRP Labelled Polymer Anti-Rabbit	Dako	K4003
Bodipy reagent	Thermo Fisher	D3922
DPBS	Gibco	14040-133
DAPI	Sigma	D9542
ProLong™ Diamond Antifade Mountant	Invitrogen	P36970
NuPAGE™ MOPS SDS Running Buffer (20X)	Invitrogen	NP000102
HEPES solution	Sigma	H0887-100ML
Tween 20	Sigma	P9616-100ML
PBS	Thermo Fischer Scientific	14040133
Tris/EDTA buffer, pH 9	Dako	S2367

Dako Real Peroxidase Blocking solution	Dako	S2023
Protein Block, Serum-free	Dako	X0909
Antibody Diluent with Background Reducing Components	Dako	S3022
Dako Wash Buffer	Dako	S3006
DAB+	Dako	K3468
Hematoxylin	Dako	S3301
Pertex	Histolab	801
TrypLE Express	Gibco	12604-021
DNase I	Sigma	DN25
Cell-ID™ 20-Plex Pd Barcoding Kit	Fluidigm	201060
Maxpar® 10X Barcode Perm Buffer	Fluidigm	201057
Maxpar® Cell Staining Buffer	Fluidigm	201068
Cell-ID™ Intercalator-Ir	Fluidigm	201192A
Maxpar® Water	Fluidigm	201069
NP-40	New England Biolabs	B2704
5% digitonin	Invitrogen	BN2006
AMPure XP beads	Beckman	A63880
BioMag® Plus Concanavalin A	Bangs Laboratories	BP531
Spermidine	Sigma	S2501
Roche Complete Protease Inhibitor, EDTA-free	Roche	5 892 791 001
PhosSTOP™	Roche	04 906 837 001
Protein-A/G-MNase	Epicypher	15-1016
EDTA	Sigma	3690
EGTA	Boston BioProducts	BM723
RNase A	Sigma	R4642
GlycoBlue	ThermoFisher	AM9515
Matrigel	Corning	356231
L-Glutamine	Sigma	G-7513
Poly-L-Lysine	Sigma	P4832
Paraformaldehyde Aqueous Solution (PFA)	Electron Microscopy Sciences	15710
L-carnitine	Sigma	C-0283
D-[^{14}C(u)]-Glucose	Perkin Elmer	NEC042A001MC
[^{14}C]-Palmitic Acid	PerkinElmer	NEC075H25OUC
Etomoxir	Sigma	E-1905-5M
NaF	Sigma	S-6776
$NaVO_4$	Aldrich	450243
Blotting-Grade Blocker	BioRad	1706404
Tris-HCl	Sigma	T2194-1L
NaCl	Sigma	S5150

$MgCl_2$	Sigma	8266
CaCl2	Sigma	C4830
Lipofectamine™ RNAiMAX Transfection Reagent	Invitrogen	13778075
Lipofectamine™ 2000 Transfection Reagent	Invitrogen	11668019
B-27™ Supplement (50X), minus vitamin A	Gibco	12587010
Recombinant Human FGF-basic (154 a.a.)	PeproTech	100-18B
hEGF	Sigma	E9644
mEGF	R&D Systems	2028-EG
DMEM/F12	Sigma	D8062
**Critical Commercial Assays**		
PI/RNase staining kit	BD Pharmigen	550825
Senescence β-Galactosidase Staining Kit	Cell Signalling	9860S
Total RNA purification Kit	NORGEN Biotek	37500
SuperScript® III First-Strand Synthesis Kit	ThermoFisher Scientific	18080-051
LightCycler® 480 SYBR Green I Master Mix	Roche	04887352001
MACH3 mouse probe	Biocare Medical	BC-M3M530H
RNA Clean & concentrator with DnaseI kit	Biosite	R1013
Nextera DNA Library Prep kit	Illumina	FC-121-1030
DNA Clean and Concentrator-5 kit	Zymo	D4014
NEBNext® Ultra™ II DNA Library Prep Kit	New Englands Biolabs	E7645
MicroScintPS	PerkinElmer	6013631
**Deposited Data**		
Sequencing data (RNA-seq, ATACseq and Cut&Run)	EMBL-EBI	PRJEB 39793
Mass cytometry data	FLOW Repository	FR-FCM-Z2TK
**Experimental Models: Cell Lines**		
MDA-MB-231	ATCC	RRID:CVCL_0062
HCC1806	ATCC	PRID:CVCL_1258
E0771	CH3 BioSystems	SKU: 94A001
TeLi	on-site	N/A
HEK293T	ATCC	PRID:CVCL_0063
**Experimental Models: Organisms/Strains**		
C57BL/6J	The Jackson Laboratory	000664
**Oligonucleotides**		
Mouse *Actin*	IDT	N/A
Forward:		
TACCACAGGCATTGTGATGG		
Reverse:		
TTTGATGTCACGCACGATTT		
Application: qPCR		

Mouse *C/ebpb*	IDT	N/A
Forward:		
GGTTTCGGGACTTGATGCA		
Reverse:		
CAACAACCCCGCAGGAAC		
Application: qPCR		
Mouse *Serpinb2*	IDT	N/A
Forward:		
TCCCAAACCTGCTACCCGAA		
Reverse:		
TGCGAGTTCACACGGAAAGG		
Application: qPCR		
Mouse *Nell2*	IDT	N/A
Forward:		
TTGGTGTGGACCCCTCCCTA		
Reverse:		
ACTTGGCGCACTCCATCTGT		
Application: qPCR		
Mouse *Cldn1*	IDT	N/A
Forward:		
TCTACGAGGGACTGTGGATG		
Reverse:		
TCAGATTCAGCAAGGAGTCG		
Application: qPCR		
Mouse *Lcn2*	IDT	N/A
Forward:		
TGCCACTCCATCTTTCCTGTT		
Reverse:		
GGGAGTGCTGGCCAAATAAG		
Application: qPCR		
Mouse *Mmp9*	IDT	N/A
Forward:		
CTTCTGGCGTGTGAGTTTCC		
Reverse:		
ACTGCACGGTTGAAGCAAAGA		
Application: qPCR		
Mouse *Serpinb7*	IDT	N/A
Forward:		
CTTCACTGCCCTGACCCTAATC		

Reverse:<br>TGCAGTGCCTTGTCAATCTGA<br>Application: qPCR		
Human *HPRT*<br>Forward:<br>CCTGACCAAGGAAAGCAAAG<br>Reverse:<br>GACCAGTCAACAGGGGACAT<br>Application: qPCR	IDT	N/A
Human *C/EBPB*<br>Forward:<br>TCGCAGGTCAAGAGCAAGG<br>Reverse:<br>TACTCGTCGCTGTGCTTGTC<br>Application: qPCR	IDT	N/A
Human *RUNX1*<br>Forward:<br>CTGCTCCGTGCTGCCTAC<br>Reverse:<br>AGCCATCACAGTGACCAGAGT<br>Application: qPCR	IDT	N/A
Human *C/EBPA*<br>Forward:<br>GGAGCTGAGATCCCGACA<br>Reverse:<br>TTCTAAGGACAGGCGTGGAG<br>Application: qPCR	IDT	N/A
Human *SERPINB2*<br>Forward:<br>CATGGAGCATCTCGTCCAC<br>Reverse:<br>ACTGCATTGGCTCCCACTT<br>Application: qPCR	IDT	N/A
Human *NELL2*<br>Forward:<br>TAAGGGTATAATGCAAGATGTCCAATT<br>Reverse:<br>AGATCTGGGCACTGAGCAATAAA<br>Application: qPCR	IDT	N/A

Human *CLADN1*<br>Forward:<br>GAAGTGCTTGGAAGACGATG<br>Reverse:<br>GAGCCTGACCAAATTCGTAC<br>Application: qPCR	IDT	N/A
Human *SERPINB7*<br>Forward:<br>CACTGGTGACTTGACCCTTCCT<br>Reverse:<br>GGTGAGACACATGGTGGTAGAATG<br>Application: qPCR	IDT	N/A
Human *CHST4*<br>Forward:<br>TGGCCATCTTGGCTCTATTC<br>Reverse:<br>CTGCTTGAAGGTCATCCACA<br>Application: qPCR	IDT	N/A
Human *MMP9*<br>Forward:<br>GTTCGACGTGAAGGCGCAG<br>Reverse:<br>TAGTGTGGTGTCTCACGAAGG<br>Application: qPCR	IDT	N/A
Human *LCN2*<br>Forward:<br>TCACCCTCTACGGGAGAACC<br>Reverse:<br>GGGACAGGGAAGACGATGTG<br>Application: qPCR	IDT	N/A
Human *LYPD6B*<br>Forward:<br>TGCAAACCTTTTCACTGTTCCA<br>Reverse:<br>GAGAGCGTGACAGAGGAGCAG<br>Application: qPCR	IDT	N/A
Human *CRISPLD1*<br>Forward:<br>TGCCCAAGAGTATACTGTCCT	IDT	N/A

Reverse:		
GATTTCGAACCACTCCAGCA		
Application: qPCR		
LCN2 siRNA	IDT	hs.Ri.LCN2.13.1, hs.Ri.LCN2.13.3
CLDN1 siRNA		hs.Ri.CLDN1.13.2, hs.Ri.CLDN1.13.3
Negative Control DsiRNA	IDT	51-01-14-04
**Recombinant DNA**		
m*C/ebpb*-shRNA-1	Sigma	SHCLNG-NM_005194, TRCN0000364533
m*C/ebpb*-shRNA-2	Sigma	SHCLNG-NM_009883, TRCN0000231409
h*C/EBPB*-shRNA-1	Sigma	SHCLNG-NM_005194, TRCN0000364533
h*C/EBPB*-shRNA-2	Sigma	SHCLNG-NM_005194, TRCN0000007443
pBABE-puro LAP2	Addgene	15712
pBABE-puro LIP	Addgene	15713
pBABE-puro	Addgene	1764
**Software and Algorithms**		
GraphPad Prism	GraphPad	Version 8.4.1
FlowJo	BD Biosciences	Version 10.7.0
R		Version 3.5.0
Cytobank		
ClonTracer	Python package	Version 1.2
**Other**		
Rat and Mouse No.1 Maintenance	Special Diet Services	RM1 (P) 801151
Rodent Diet With 60 kcal% Fat	Research Diets	D12492

Uncropped blots from Supplementary Figure 4 a

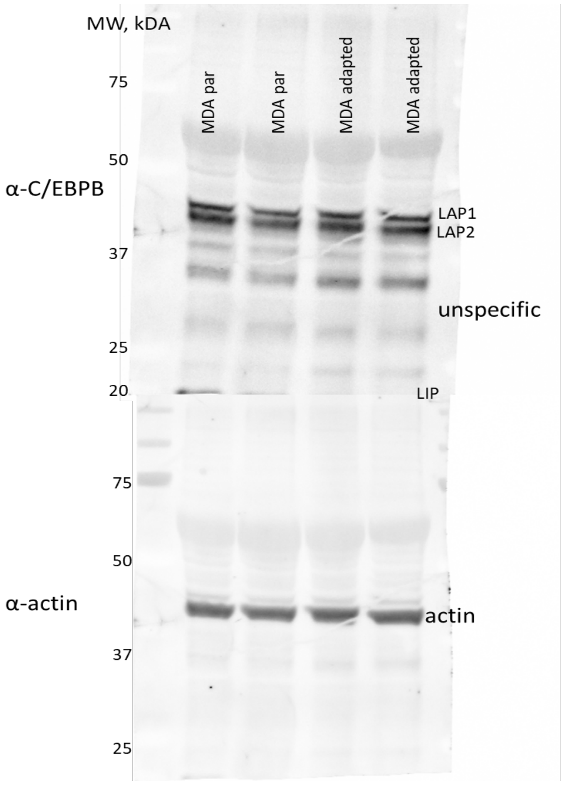

Uncropped blots from Supplementary Figure 4 b

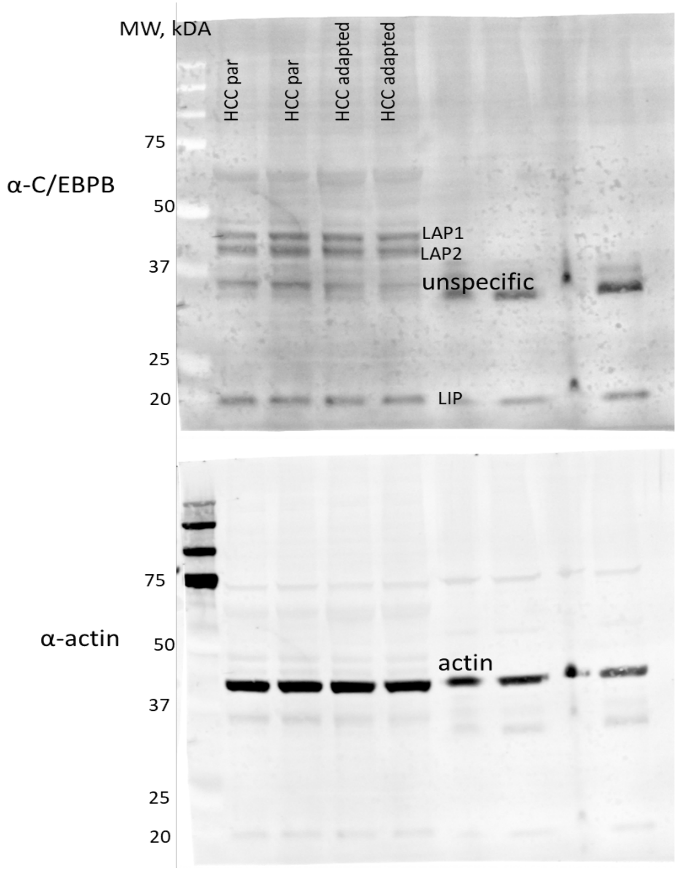

Uncropped blots from Supplementary Figure 4 c

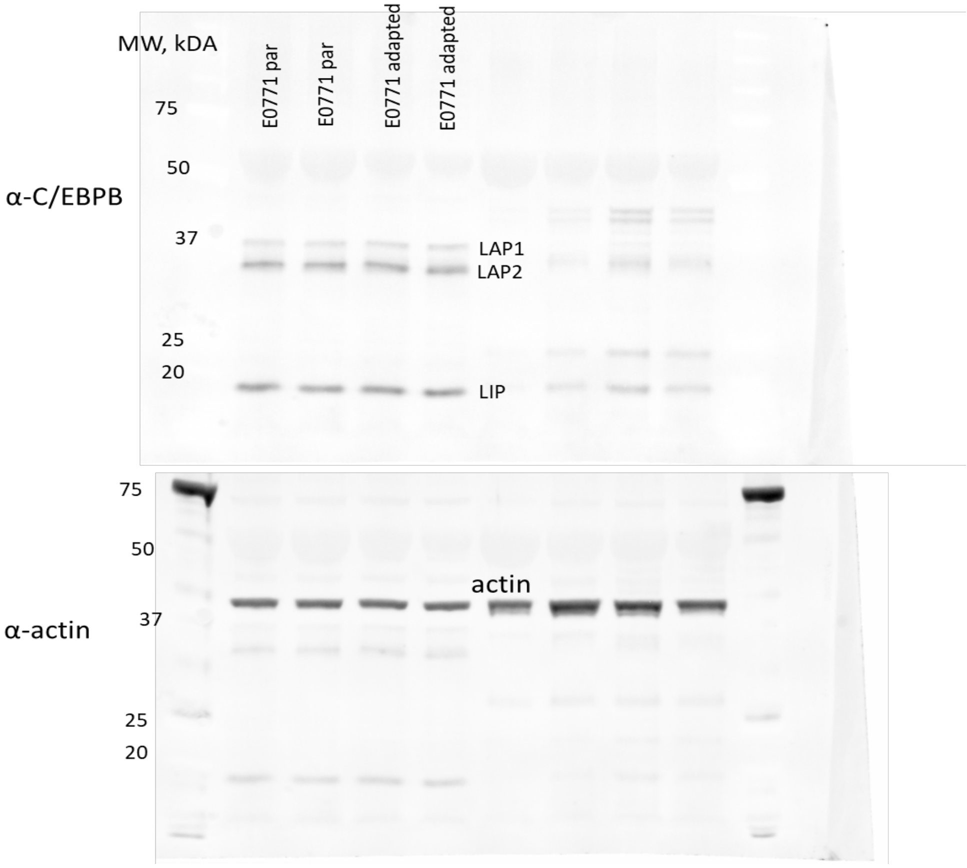

Supplement: Supplementary file 1 — Supplementary Information [file 41467_2021_27734_MOESM1_ESM.pdf]
