# Supplementary material for: C/EBPB-dependent adaptation to palmitic acid promotes tumor formation in hormone receptor negative breast cancer
Source: Nat Commun. 2022 Jan 10;13:69. doi: 10.1038/s41467-021-27734-2 (PMC8748947; doi:10.1038/s41467-021-27734-2)
Supplement: Supplementary file 3 — Reporting Summary [file 41467_2021_27734_MOESM3_ESM.pdf]

nature portfolio

Corresponding author(s): Nils Halberg

Last updated by author(s): Nov 30, 2021

Reporting Summary

Nature Portfolio wishes to improve the reproducibility of the work that we publish. This form provides structure for consistency and transparency in reporting. For further information on Nature Portfolio policies, see our Editorial Policies and the Editorial Policy Checklist.

Statistics

For all statistical analyses, confirm that the following items are present in the figure legend, table legend, main text, or Methods section.

n/a	Confirmed	
☐	☒	The exact sample size (*n*) for each experimental group/condition, given as a discrete number and unit of measurement
☐	☒	A statement on whether measurements were taken from distinct samples or whether the same sample was measured repeatedly
☐	☒	The statistical test(s) used AND whether they are one- or two-sided<br>*Only common tests should be described solely by name; describe more complex techniques in the Methods section.*
☒	☐	A description of all covariates tested
☐	☒	A description of any assumptions or corrections, such as tests of normality and adjustment for multiple comparisons
☐	☒	A full description of the statistical parameters including central tendency (e.g. means) or other basic estimates (e.g. regression coefficient) AND variation (e.g. standard deviation) or associated estimates of uncertainty (e.g. confidence intervals)
☐	☒	For null hypothesis testing, the test statistic (e.g. *F*, *t*, *r*) with confidence intervals, effect sizes, degrees of freedom and *P* value noted<br>*Give P values as exact values whenever suitable.*
☒	☐	For Bayesian analysis, information on the choice of priors and Markov chain Monte Carlo settings
☒	☐	For hierarchical and complex designs, identification of the appropriate level for tests and full reporting of outcomes
☒	☐	Estimates of effect sizes (e.g. Cohen's *d*, Pearson's *r*), indicating how they were calculated

Our web collection on statistics for biologists contains articles on many of the points above.

Software and code

Policy information about availability of computer code

Data collection

- Mass cytometry samples were measured with Helios Mass Cytometer
- Flow cytometry samples were measured with BD FACSDIVA and BD Fortessa
- Tissue microarray slides were scanned with an Aperio Scanscope CD SLide Scanner
- RNAseq and ATACseq damples were sequenced on Illumina HiSeq4000
- Cut&Run samples were sequenced on Illumina MiSeq
- ClonTracer barcoded samples were sequenced on Illumina MiSeq
- Western Blots were acquired on Amersham Typhoon gel and Blot imaging Systems
- Fluorescence imaging was performed on SP5 Leica
- Proliferation assays were done using Incucyte Zoom (v2018A)
- Brighfield imaging was done on Nikon TE2000 microscope
- qPCR was performed on LightCycler 480 Instrument II
- Microarray data was obtained on Illumina BeadArray Reader

Data analysis

- Mass cytometry raw FCS files were normalized, concatenated and debarcoded in R using CATALYST (v3.10). Samples from teh different conditions were downsampled (85000 for MHCC1806 and 100000 for MDA-MB-231) prior analysis using Cytobank. Dimensionality reduction with tSNE, density plotting and pseudo coloring were performed in Cytobank.
Flow cytometry data were analyzed in FloJo
- For tissue microarray analysis, QuPath (v0.2.0-m5) was used to dearray the TMAs, segment cells and classify cell type.
- Microarray beadrays were analyzed using the Illumina Bead Scan Software.
- Software and algorithms used for bioinformatics: Sequenced reads were quality checked with FastQC (v0.11.9). Adapter sequences were trimmed using Trimmomatic. The trimmed reads were aligned to the UCSC hg19 reference genome using Bowtie2. Differentially expressed genes were identified using DESeq2 (v1.34). For ATACseq data, MACS2 was used for peak calling and deepTools (v3.3.1) was used to generate

1x nominalized bigwig files for visualization. Analysis of differentially accessible peak was performed using DiffBind, and annotated genome-wide with respect to the closest transcriptional start site with ChIPSeeker. To infer differential transcription factor binding motif activity, difTF (v1.4) was used. For Cut&Run data, peaks were called with SEACR. Consensus peaksets across samples were generated using DiffBind (v 3.0), and annotated genome-wide with respect to the closest transcription start site with ChIPSekker. For motif discovery within peaks, EChO was run to identify direct binding sites in the foci mode. Homer was used for motif enrichment analysis. Footprints were detected using Footprint Occupancy Score (FOS).
- barcode-composition analysis was carried out by using the python package clonTracer v1.2
-Immunofluorescence images were analyzed by FIJI.

For manuscripts utilizing custom algorithms or software that are central to the research but not yet described in published literature, software must be made available to editors and reviewers. We strongly encourage code deposition in a community repository (e.g. GitHub). See the Nature Portfolio guidelines for submitting code & software for further information.

Data

Policy information about availability of data

All manuscripts must include a data availability statement. This statement should provide the following information, where applicable:
- Accession codes, unique identifiers, or web links for publicly available datasets
- A description of any restrictions on data availability
- For clinical datasets or third party data, please ensure that the statement adheres to our policy

All data generated and analysed during this study are available within the Article and Supplementary Files, or available from the authors upon request. Sequencing data (RNA-seq, ATAC-seq, Cut&Run and ClonTracer barcode assay) for this study have been deposited in the European Nucleotide Archive (ENA) at EMBL-EBI under accession number PRJEB 39793. The mass cytometry data has been deposited in the FLOWRepository under repository ID FR-FCM-Z2TK.

Field-specific reporting

Please select the one below that is the best fit for your research. If you are not sure, read the appropriate sections before making your selection.

☒ Life sciences ☐ Behavioural & social sciences ☐ Ecological, evolutionary & environmental sciences

For a reference copy of the document with all sections, see nature.com/documents/nr-reporting-summary-flat.pdf

Life sciences study design

All studies must disclose on these points even when the disclosure is negative.

Sample size	Sample size was determined based on what is commonly used in the field. For TMA experiment, all available samples that passed quality control (Cores with too few cells, poor quality, excessive tearing, or folding were not considered valid and were omitted from analysis) were included. For in vivo experiments, at least 5 mice per group were used. For mass cytometry, 85 000 cell per replicate was used. All samples were downsampled to match the number in the sample in which the event count was lowest using Cytobank.
Data exclusions	For Axl TMA experiment, an outlier value deviating more than 2 times the standard deviation in the Axlhigh BMI under 25 group was excluded prior to statistics performed.
Replication	All in vitro functional assays are conducted with replication in at least three independent experiments to confirm reproducibility. For all in vivo work sample sizes were determined at the start of each cohort. We performed multiple experiments with varying cohort sizes to strengthen the reproducibility of the data. The experiments required significant starting material for downstream applications, therefore it was necessary to perform several experiments with smaller n, but following the exact same protocol. In all in vivo cohorts the data were highly reproducible. The fact that our data and observations were reproducible in the separate cohorts, gave us confidence that our sample size is adequate.
Randomization	For in vivo experiments, female littermates were randomly assigned to chow and HFD groups. For in vitro experiments, cell lines were cultured in the same conditions and randomly divided into different conditions for experiments.
Blinding	Measurements for all animal experiments were conducted in a blinded manner. For in vitro experiments, investigators were not blinded during data collection and analysis because experimental set up involved visible identification labels of different conditions. Data analysis was performed and confirmed by multiple investigators. For TMA experiment, the quality control of TMA samples were done in a blinded manner.

Reporting for specific materials, systems and methods

We require information from authors about some types of materials, experimental systems and methods used in many studies. Here, indicate whether each material, system or method listed is relevant to your study. If you are not sure if a list item applies to your research, read the appropriate section before selecting a response.

Materials & experimental systems

n/a	Involved in the study
	☒ Antibodies
	☒ Eukaryotic cell lines
☒	☐ Palaeontology and archaeology
	☒ Animals and other organisms
	☒ Human research participants
☒	☐ Clinical data
☒	☐ Dual use research of concern

Methods

n/a	Involved in the study
☒	☐ ChIP-seq
	☒ Flow cytometry
☒	☐ MRI-based neuroimaging

Antibodies

Antibodies used

Primary antibodies for Western Blot:
- C/EBPB, Santa Cruz, sc-7962
- Beta-actin, Invitrogen, PA1-183
Secondary Antibodies for Western Blot
IRDye® 800CW Donkey anti-Rabbit IgG (H + L), 0.1 mg Leicor [P/N 926-32213], 0,1 mg
IRDye® 680RD Goat anti-Mouse IgG (H + L), 0.1 mg Leicor [P/N 925-68070], 0,1 mg
Antibodies for Cut&Run:
- C/EBPB, Santa Cruz, sc-7962
- Mouse IgG isotype, Merck-Millipore, 12-371
- Anti-Histone H3 (mono methyl K4) antibody - ChIP Grade, Abcam, ab8895
- Anti-trimethyl-Histone H3 (Lys27) Antibody, Merck, 07-449
- Rabbit IgG, Diagenode, C15410206
Antibody for TMA staining:
- CD133, Miltenyi Biotec, 130-090-422
- Axl, R&D Systems, AF154
Antibodies for Flow Cytometry:
- CD133-APC, Invitrogen, 17-1331-81
- #D44-FITC, BioLegend, 338803
Antibodies for Mass Cytometry:
- E-cadherin-158Gd, Fluidigm, 3158021A
- Cleaved caspase 3-142Nd, Cell Signalling technology, Clone SA1E
- CD44-173Yb, Fluidigm, 3150018B
- CD133-160Gd, Miltenyi Biotec, 130-090-422
- Axl-168Er, BGB/creative biolabs, HPAB-0110-LS
- pEGFR-151Eu, Abcam, ab32430
- pCreb-176Yb, Fluidigm, 3176005A
- pAkt-152Sm, Fluidigm, 3156002A
- P38-156Gd, Fluidigm 3156002A
- N-cadherin-143Nd, Fluidigm, 3143016B
- Keratin7-164Dy, BD, ab9021
- EGFR-170Er, Fluidigm, 3170009B
- YAP-167Er, Santa Cruz, sc-271134
- Vimentin-154Sm, Fluidigm, 3154014A
- TGF"-163Dy, Fluidigm, 3163010B
- pStat5-147Sm, BD, 562077
- pStat3-145Nd, BD, 624084
- pStat1-153Eu, Fluidigm, 3153005A
- pSHP2-141Pr, Fluidigm, 3141002A
- pS6-172Yb, Fluidigm, 3172008A
- PRb-150Nd, Fluidigm, 3150013A
- pNFKB-166Er, Fluidigm, 3166006A
- pMAPKAPK2-159Tb, Fluidigm, 3159010A
- pHistone H3-175Lu, Fluidigm, 3175012A
- pErk1/2-171Yb, BD, 624084
Primary antibodies for IF:
- C/EBPB, Santa Cruz, sc-7962
Secondary Antibodies for IF:
- AF647, goat anti-mouse Life Technologies, A21238

Validation

All antibodies were validated by the manufacturer (typically cell type specific staining patterns) and are widely used in the field. C/EBPB antibody was validated using knockdown and over expression experiments. Validation and titration experiments were performed for all antibodies used mass cytometry experiment. Also, after completion of the experiment, antibody specificity was confirm by comparisons of cell specific markers across the high dimensional data set.

Eukaryotic cell lines

Policy information about cell lines

Cell line source(s)	Human cell lines (MDA-MB-231, HCC1806 and HEK293T cells) were obtained from ATCC. Mouse cancer cell line (E0771 cells) was obtained from CH3 BioSystems. TeLi (basal breast cancer, mouse) cells were originally derived from a tumor formed in MMTV-Wnt1 transgenic mouse. Tumors was dissociated using Mouse tumor dissociation kit (Miltenyi Biotec, 130-096-730). Dissociated tumor cells were cultured in vitro for two months to obtain pure tumor cells.
Authentication	For cell line authentication, 2*10^6MDA-MB-231 and HCC1806 cells were collected, resuspended in 500 µl 70% Ethanol and sent to Microsynth for cell line authentication and the cell lines were authenticated based on genetic fingerprinting and short tandem repeat (STR) profiling.
Mycoplasma contamination	All cell lines are routinely tested for mycoplasma contamination and cells used in this study are negative for mycoplasma contamination.
Commonly misidentified lines (See ICLAC register)	No cell line used in this paper is listed in ICLAC database.

Animals and other organisms

Policy information about studies involving animals; ARRIVE guidelines recommended for reporting animal research

Laboratory animals	C57BL/6J mice were obtained from Jackson Laboratories and bred on site. 6 weeks old female littermates were randomly assigned to chow and HFD groups and fed either standard chow diet (7.5% kcal from fat, 17.5% from proteins and 75% from carbohydrates, Special Diet Services RM1, 801151) or high fat containing diets (60% kcal from fat, 20% from protein and 20% from carbohydrates, Research Diets, D12492) for 10 weeks prior to tumor cell implantations.
Wild animals	No wild animals were used
Field-collected samples	This study did not involve field-collected samples
Ethics oversight	Experiments were approved by the Norwegian Food Safety Authority (FOTS ID 10101 and 24723)

Note that full information on the approval of the study protocol must also be provided in the manuscript.

Human research participants

Policy information about studies involving human research participants

Population characteristics	Patients were female breast cancer patients aged 25-70 years old with known BMI (between 19 and 38 kg/m2). Samples were collected at diagnosis prior to treatment. All patients were treated with epi-adriamycin or paclitaxol.
Recruitment	Patients were recruited at the Haukeland University Hospital and the hospitals in Oslo (Skt Olav), Tromsø, Trondheim and Stavanger. The trial recruited patients with primary stage III breast cancers in Norway, from 1997 to 2003. Norway has (and had, at the time of recruitment) a public health care system treating close to 100% of breast cancer cases. As such, there is no socioeconomic bias in the data set from patients recruited from trials run in the public health care system. The trial recruited 223 patients. Out of these, expression arrays were run for 203. This selection was solely based on the availability of remaining material (biopsy tissue for RNA-isolation). As such, the selection for expression array did not introduce any biologically relevant bias in the data.
Ethics oversight	The study was approved by the regional committees for medical and health research of Western Norway (REK-Vest; approval number 273/96-82.96)

Note that full information on the approval of the study protocol must also be provided in the manuscript.

Flow Cytometry

Plots

Confirm that:

- [x] The axis labels state the marker and fluorochrome used (e.g. CD4-FITC).
- [x] The axis scales are clearly visible. Include numbers along axes only for bottom left plot of group (a 'group' is an analysis of identical markers).
- [x] All plots are contour plots with outliers or pseudocolor plots.
- [x] A numerical value for number of cells or percentage (with statistics) is provided.

Methodology

Sample preparation	For immunostaining for flow cytometry, cells were collected using Accutase (Sigma, A6964) and washed once in PBS. 1×106

	cells per sample were stained with 0.6 µL of APC conjugated CD133 antibody (Invitrogen, 17-1331-81) and 1 µL of FITC conjugated CD44 antibody (Biolegend, 338803) in 1000ul of 1%BSA supplemented PBS solution and incubated in the dark for 20 mins at room temperature. After incubation, cells were washed once in 5ml PBS/1% BSA and analyzed by flow cytometry
Instrument	BD Fortessa
Software	Acquired data were analyzed by FlowJo v10.7.0
Cell population abundance	Flow analysis were done on in vitro propagated cancer cells. All events were recorded.
Gating strategy	FSC-A and SSC-A were used to exclude debris and dead cells. FSC-A and FSC-A and FSC-H were used to exclude doublets. To gate the CD44high/CD133+ cell populations, the mean fluorescent intensity (MF)) of CD44-FITC was measured on control replicates (termed parental cells in Supplemental Fig S2F-G and overexpression control cells in Supplementary Fig S4E-F)The average value of CD44-FITC MFI was used to gate CD44high cells, and CD133+ cells were gated according to the negative staining samples

☒ Tick this box to confirm that a figure exemplifying the gating strategy is provided in the Supplementary Information.
